# Risk stratification using data from electronic medical records better predicts suicide risks than clinician assessments

**DOI:** 10.1186/1471-244X-14-76

**Published:** 2014-03-14

**Authors:** Truyen Tran, Wei Luo, Dinh Phung, Richard Harvey, Michael Berk, Richard Lee Kennedy, Svetha Venkatesh

**Affiliations:** 1 Centre for Pattern Recognition and Data Analytics, Deakin University, Geelong 3220, Australia; 2 Department of Computing, Curtin University, Bentley, Australia; 3 Mental Health Services, Barwon Health, Geelong, Australia; 4 School of Medicine, Deakin University, Geelong, Australia; 5 Barwon Health, Geelong, Australia; 6 Mental Health Research Institute, University of Melbourne, Parkville, Australia; 7 Orygen Youth Health Research Centre, Parkville, Australia

**Keywords:** Suicide risk, Electronic medical record, Predictive models

## Abstract

**Background:**

To date, our ability to accurately identify patients at high risk from suicidal behaviour, and thus to target interventions, has been fairly limited. This study examined a large pool of factors that are potentially associated with suicide risk from the comprehensive electronic medical record (EMR) and to derive a predictive model for 1–6 month risk.

**Methods:**

7,399 patients undergoing suicide risk assessment were followed up for 180 days. The dataset was divided into a derivation and validation cohorts of 4,911 and 2,488 respectively. Clinicians used an 18-point checklist of known risk factors to divide patients into low, medium, or high risk. Their predictive ability was compared with a risk stratification model derived from the EMR data. The model was based on the continuation-ratio ordinal regression method coupled with lasso (which stands for least absolute shrinkage and selection operator).

**Results:**

In the year prior to suicide assessment, 66.8% of patients attended the emergency department (ED) and 41.8% had at least one hospital admission. Administrative and demographic data, along with information on prior self-harm episodes, as well as mental and physical health diagnoses were predictive of high-risk suicidal behaviour. Clinicians using the 18-point checklist were relatively poor in predicting patients at high-risk in 3 months (AUC 0.58, 95% CIs: 0.50 – 0.66). The model derived EMR was superior (AUC 0.79, 95% CIs: 0.72 – 0.84). At specificity of 0.72 (95% CIs: 0.70-0.73) the EMR model had sensitivity of 0.70 (95% CIs: 0.56-0.83).

**Conclusion:**

Predictive models applied to data from the EMR could improve risk stratification of patients presenting with potential suicidal behaviour. The predictive factors include known risks for suicide, but also other information relating to general health and health service utilisation.

## Background

Suicidal ideation occurs in more than 10% of the population during their lifetime [1]. Each year, 2% of the population contemplate suicide and 0.3% attempt suicide [2]. Across all age groups, the incidence and prevalence of suicidal behaviour have increased considerably over the past two decades. Suicide is now a more common cause of death than motor vehicle accidents [3-5]. Suicide does not happen without warning. People frequently make contact with health services in the months leading up to their suicide attempt. There is a recognised need to identify those at risk [6,7] for targeted interventions to stop suicide before it happens [8].

Risk factors for suicide are well-documented, but the list is long. These include: age, male gender, chosen method of attempted suicide, and number of previous attempts; [9] psychiatric diagnoses including anxiety and depression, psychosis, and bipolar disorder; [10-12] social isolation; [13] and potential lethality of previous attempts [14]. Despite the effort to combine these risk factors into risk scores and algorithms to predict suicide risk, [15-17] the predictions are often too poor to be clinically useful [18,19].

Alternative to a constant set of risk scores that we hope would work for each individual, a broader source of information is available: the comprehensive electronic medical record (EMR). The time-stamped administrative, clinical, and investigative data in the EMR includes information on known risk factors for suicide. Additionally, it contains information on health service encounters that might not be directly related to suicidal behaviour. It is known that patients who attempt suicide frequently attend the emergency department (ED) in the months before their suicide attempt [20,21]. Many of these attendances are not directly related mental health problems or self-harm. Also, many suicidal patients attend primary care facilities in the months before their suicide attempt, again commonly for reasons not directly related to their psychological distress [22]. Up to 85% of suicidal patients attend hospital outpatients in the twelve months before their suicide attempt, [23] again often for reasons not directly related to psychological morbidity. There is a high prevalence of coexistent physical illnesses amongst patients who exhibit suicidal behaviour [24]. All these contextual factors may contribute to suicide risk, but for each individual patient, they are difficult to be assessed objectively and consistently without a proper tool.

We develop a statistical risk stratification model based on EMR data. The model results from examining suicide attempts and completed suicide in a large cohort of patients who underwent suicide assessment in a regional health service. We compare EMR-based predictions of high-risk suicidal behaviour with clinician predictions, which are based on an 18-point assessment instrument.

## Methods

### Study design and population

This was a retrospective study using electronic records of inpatient admissions and emergency department (ED) visits within Barwon Health, a regional health service in Australia. As the only tertiary hospital in Greater Geelong, and a catchment area with over 350,000 residents, the hospital’s patient database provides a single access point for information on hospitalisations, ED visits, and medications. Although different IT systems are used by hospitals, EMRs often share similar underlying logical structure. All the diagnoses are often coded using the data standard ICD-10. For generalisability of the model, we focus on EMR data that are either generally available in mental health services or coded using the ICD-10 disease classification.

Patients were included in the study if they were aged 10 years or over, and had received at least one suicide risk assessment between April 2009 and March 2012. A cohort of 7,399 patients (with 16,858 risk assessments) met the inclusion criteria. Risk assessments were carried out either in Barwon Health following presentation to ED or another department or in one of five community health centres. Patient outcomes were observed for a 180 day period following a risk assessment and details of repeat suicide attempts or completed suicides were recorded. A risk assessment was considered a clinically relevant time point for prediction, as care models are devised according to patient risk at that time. If a patient had more than one assessments, one of them would be randomly selected.

Patient records prior to the selected assessment date were used to construct the independent variables. The same data were also available to clinicians during risk assessments. Suicide attempts, of varying lethality, in a 180 day period following the assessment were treated as the dependent variable. The true outcome, for a given period, is the actual risk class determined from patient records for that period. As outlined before, direct modelling of suicide risk is difficult because of low base rate of suicides. Modelling risk of suicide attempts, a strong predictor of suicide, [25] is a pragmatic measure to circumvent this difficulty. Moreover, it allowed us to define a set of risk classes for the suicide attempts of varying lethality levels, consistent with previous published work [26].

Self-harm events are recorded in the hospital database using ICD-10 codes. For example, an ED visit due to by attempted hanging, would be assigned the ICD-10 code X70. We exclude those events which may result from accidents or assaults (instead of intentional self-harm). In Australia, such events are coded with the following ICD-codes: Transport Accidents (V01-V99, Y85); Falls (W00-W19); Accidental Poisoning (X40-X49); Assault (X85-Y09,Y87.1).

For each stipulated period following a risk assessment, the risk class was defined as follows:

1. The period has high-risk if, during the period, the patient had an ED visit or inpatient admission with a high-lethality diagnosis (Table 1).

**Table 1:** ICD-10 codes identified to correlate with moderate or high lethality events

** ICD-10 codes**	**Diagnosis**
**Moderate lethality**	
F04	Organic amnesic syndrome
F05.0, F05.8, F05.9	Delirium
F10.0, F10.6, F11.x-F16.x, F18.x, F19.x	Mental disorders due to alcohol and drugs
F63.1, F63.2	Pyromania and kleptomania
S00.x, S01.x, S02.2-S02.6, S03.0, S10.0-S10.8, S11.x, T00.3-T00.9, W25, W26, Y28, Y29	Superficial injuries
T40.7-T40.9, T42.4, T42.8, T43.2, T43.5, T44.2-T44.5, T44.9, T45.0, T45.1, T51.x, T52.1-T52.4, T52.9, T53.1-T53.9, T60.8, T60.9, T62.0, T62.1, T65.3, Y10, Y11, Y13-Y19	Poisoning, moderate severity
X60, X61, X65, X78, X79, X83, X84, Y87.0	Intentional self-harm, not life-threatening
Y33, Y34, Y86	Event of undetermined intent
Y90.1-Y90.4, Y91.0-Y91.2, Y91.9	High alcohol level in blood
Z91.5	Personal history of self-harm
**High lethality**	
S02.0, S02.1, S02.7-S02.9, S06.x-S09.x, S12.x, S13.0-S13.4, S17.x-S19.x, S21.1, S21.8, S21.9	Severe injuries
T40.0-T40.6, T42.3, T42.5-T42.7, T43.1, T43.1, T43.3, T43.4, T43.6-T43.9, T44.0, T44.1, T44.8, T46.x, T51.3, T52.0, T52.8, T53.0, T54.x, T56.1, T57.3, T58, T59.2, T59.4, T59.5, T60.4, T65.0, T65.1	Severe poisoning
T71	Asphyxiation
T73.2	Exhaustion due to exposure
T75.1, W65-W74	Drowning and nonfatal submersion
T75.4	Effects of electric current
X62-X64, X66-X77, X80-X82	Intentional self-harm and self-poisoning
Y12, Y20-Y27, Y30-Y32	Event of undetermined intent
Y90.5-Y90.8, Y91.3	Very high alcohol level in blood

2. The period has moderate-risk if, during the period, the patient had an ED visit or inpatient admission with a moderate-lethality diagnosis (Table 1).

3. Otherwise, the period has a low-risk (patients with low lethality suicide attempts or no suicide attempts).

Predictive algorithms were constructed from the derivation cohort to predict risk in different post-assessment periods—30, 60, 90, and 180 days. The predictive model takes demographic and historical data prior to a risk assessment, and identifies the post risk-assessment class, for a desired period.

Ethics approval was obtained from the Hospital and Research Ethics Committee at Barwon Health, with whom Deakin University has reciprocal ethics authorisation (approval number 12/83).

### Clinician risk assessment

The clinicians’ prediction of risk involves using a risk assessment checklist. Clinical protocols at Barwon Health require a suicide risk assessment to be completed on intake to care, every 91 days during an episode of care and on discharge. For the purpose of this study all available suicide risk assessment data was extracted from the data warehouse. The primary purpose of the assessment is to ensure that consideration of suicide risk is medico-legally documented. Under the Victorian Mental Health Act, mental health services are required to report deaths of patients who are either currently receiving care or have had contact with the service in the past six months. Patient deaths are reported through a centralised risk management system, which is also reconciled against death certificates and a registry of deaths. Deaths reported to the coroner are also tracked to the point where the coroner determines whether the death was due to suicide. All suicides and other unnatural deaths are subject to a comprehensive case review. Data on self-harm was identified by diagnostic codes for intentional overdoses and self-inflicted injuries from admission and emergency presentation coding. The suicide risk assessment instrument used was developed by Barwon Health in 1999, based on known risk factors in the literature. It has been in use for more than 13 years as no alternative risk scores have been shown to be more effective. One of the purposes of this study was to validate use of this risk assessment tool. The tool consists of 18 items, each graded on a 3-point scale (low/moderate/high) covering suicidal ideation, suicide plan, access to means, prior attempts, anger/hostility/impulsivity, depression (current level), anxiety, disorientation/disorganisation, hopelessness, identifiable stressors, substance abuse, psychosis, medical status, withdrawal from others, expressed communication, psychiatric service history, coping strategies and supportive others (connectedness).

### Derived predictor variables

Patient specific information—such as interactions with health services, ICD-10 diagnostic codes (mental disorders and other comorbidities), diagnosis-related groups (DRGs) and procedures, demography, and medications—were used to derive predictors. Diagnostic codes were used to derive following subsets: injuries classified as moderate or high risk, mental diagnoses mapped into Australia’s Mental Health Diagnostic Groups, and relevant codes mapped into Elixhauser comorbidities [27]. To ensure generalisability, medications were mapped according to the World Health Organisation’s Anatomical Therapeutic Chemical (ATC) classification system. Different from most existing clinical predictive models, derived predictors are specific in the temporal dimension, encoding the knowledge that prior events may influence future risks at multiple time scales. More specifically, occurrences of historical data were aggregated and normalised over several time periods before the assessment point: 0–3 months, 3–6 months, 6–12 months, 12–24 months, and 24–48 months. Overall 5,322 variables were derived, but only those that appeared more than 4 times among the patients at risk in the derivation cohort were kept, resulting in 202 variables. For robustness, variable values outside the 1^st^ and the 99^th^ percentiles were considered outliers and rounded to the nearest percentiles.

### Derivation of the risk stratification model

For predictive modelling, we adopted a recently established statistical technique *L1-penalised continuation-ratio model for ordinal outcomes*[28]. Different from traditional ordinal regression models that assume monotone effects of predictor variables [29], the continuation-ratio model uses separate coefficients for different outcome classes, at the same time automatically selects highly predictive variables (and their time scales) for each class. As prior studies suggest that predictors of the two classes may be different [30], it is desirable to model the moderate-risk and the high-risk classes using separate coefficients. The variable selection capability comes from the lasso-like shrinkage [31] that forces variables with weak association with the outcomes to have zero coefficients. The shrinkage strength was adjusted so that the predictive performances on the derivation and validation cohorts were similar, and thus ensuring no over-optimism in the estimated model [32].

A single prediction risk score was computed, representing the expected lethality level. The risk score takes a value from 0 to 2, with 0, 1, and 2 representing low, moderate, and high risk respectively. This score results in a simple decision rule: Given two thresholds in the range 0 – 2, the outcome class is low-risk if the score is less than the lower threshold, moderate-risk if the score is below the upper threshold and high-risk otherwise. The thresholds can be adjusted according to the clinical need.

The contribution of individual risk factors toward each risk class was assessed using bootstrap. That is, the models were estimated multiple times from bootstrap samples, and variable coefficients were then collected and statistics were derived. Among them are Wald statistics, confidence intervals, variable importance, and selection probability. Variable importance is defined as the product of absolute mean coefficient and variable standard deviation. Selection probability is the chance that a variable is being selected by applying the lasso shrinkage.

### Model validation

To assess the performance of prediction methods, the 7,399 patients were randomly divided into a derivation cohort of 4,911 patients and a validation cohort of 2,488 patients. The risk score assigned by the predictive model and clinician-assigned overall risk were validated against true outcomes on the validation cohort. The risk score makes it simple for estimating performance measures for multiple binary decisions such as high-risk versus the rest, or moderate/high-risk versus low-risk. We reported here Area under the ROC Curves (AUC), sensitivity and specificity. The confidence intervals for these measures were estimated using bootstrap.

## Results

### Patients

Characteristics of patients included in the study are summarised in Table 2. In the follow up periods of 30 days, 90 days, and 180 days, there were 7, 9, and 13 suicides. The mean age was 41.2 years (11 – 101 years). Overall, 2,479 (33.5%) patients had changed postcode in the preceding 12 months. 3,953 (53.2%) attended ED in the 3 months before suicide assessment, and 4,436 (60.0%) had at least one hospital admission during the preceding 12 months. Of the 7,399 patients, 1,562 (21.1%) had a moderate lethality suicide attempt in the 12 months preceding their assessment and 646 (8.7%) patients had a high lethality attempt. A further breakdown of factors according to the outcomes within 90-day is presented in Table 3. Overall, risk factors were more prevalent in the more risky groups. The prevalence of males, pensioners, prior moderate-risk events, past 3-month admission and alcohol abuse were highest in the moderate-risk group. Other risk factors were most prevalent in the high-risk group.

**Table 2:** Demographic and clinical characteristics

**Feature**	**Number (%)**
Total number of patients	7,399 (100)
Age	
≤ 20	1,213 (16.4)
21–35	2,071 (28.0)
36–55	2,080 (28.1)
56–70	674 (9.1)
≥ 71	981 (13.3)
Male	3,649 (49.3)
Divorced/separated	811 (11.0)
Unemployed/home duties	1,235 (16.7)
Pensioner/retired	1,417 (19.2)
**Postcode changed**	
Past 12 months	2,479 (33.5)
Past 12–24 months	1,201 (16.2)
Past 24–48 months	1,802 (24.4)
**Admitted before risk assessment**	
Past 3 months	1,824 (24.7)
Past 3–6 months	1,127 (15.2)
Past 6–12 months	1,485 (20.0)
**ED visited before risk assessment**	
Past 3 months	3,935 (53.2)
Past 3–6 months	1,297 (17.5)
Past 6–12 months	1,681 (22.7)
**Attempted before risk assessment - moderate lethality**	
Past 3 months	817 (11.0)
Past 3–6 months	310 (4.2)
Past 6–12 months	435 (5.9)
**Attempted before risk assessment - high lethality**	
Past 3 months	369 (4.0)
Past 3–6 months	120 (1.6)
Past 6–12 months	157 (2.1)

**Table 3:** Risk factors within outcome groups (low, moderate and high-risk events) in 90-days

	***Low (n = 7,002)***	***Moderate (n = 247)***	***High (n = 150)***
**Demographics**			
Male (%)	48.9	64.4	45.3
Divorced/separated (%)	10.8	12.2	18.7
Unemployed (%)	7.2	14.6	15.3
Pensioner/retired (%)	18.9	25.5	19.3
Postcode change in past 12 months (%)	33.0	39.3	46.7
**Admitted before risk assessment**			
In past 3 months (%)	23.7	45.3	36.0
In past 3–6 months (%)	14.6	25.5	27.3
In past 6–12 months (%)	19.3	37.3	29.3
**ED visited before risk assessment**			
In past 3 months (%)	52.0	74.5	74.7
In past 3–6 months (%)	16.8	30.4	32.0
In past 6–12 months (%)	21.9	37.3	38.0
**Prior moderate lethality risk event**			
In past 3 months (%)	9.9	31.6	29.3
In past 3–6 months (%)	3.7	15.0	9.3
In past 6–12 months (%)	5.0	22.7	18.0
**Prior high lethality risk event**			
In past 3 months (%)	4.5	7.7	24.0
In past 3–6 months (%)	1.5	2.0	8.0
In past 6–12 months (%)	1.9	6.5	7.3
**Prior mental health within 12 months**			
Alcohol abuse (%)	4.6	27.1	15.3
Depressive episode (%)	5.4	9.3	14.0

### Validation of the 18-point risk score

Risk scores from the 18-point risk assessment instrument with respect to 90-day outcomes are shown in Table 4. Overall most identified factors were scored higher in the high risk group. Not surprisingly, the differential between low and high risk groups was highest for the factor ‘prior attempt’ (the average score for the high risk group was 1.70 compared to 1.26 for the low risk).

**Table 4:** Average risk scores assigned to 18-item checklist within true outcome groups (low, moderate and high-risk events) in 90-days

***Item***	***Low***	***Moderate***	***High***
Suicidal ideation	1.08	1.08	1.30
Suicide plan	1.04	1.05	1.25
Access to means	1.15	1.22	1.39
Prior attempts	1.26	1.34	1.70
Anger/Hostility/Impulsivity	1.27	1.38	1.42
Depression (current level)	1.29	1.26	1.40
Anxiety	1.37	1.33	1.44
Disorientation/Disorganisation	1.04	1.14	1.08
Hopelessness	1.30	1.32	1.51
Identifiable stressors	1.61	1.62	1.87
Substance abuse	1.22	1.58	1.44
Psychosis	1.01	1.13	1.04
Medical status	1.20	1.34	1.35
Withdrawal from others	1.20	1.24	1.31
Expressed communication	1.08	1.19	1.21
Psychiatric service history	1.08	1.23	1.21
Coping strategies	1.25	1.40	1.51
Supportive others (connectedness)	1.35	1.45	1.56

### Comparison of clinician and machine model predictions

To assess the performance of the predictive model using EMR data, we compared it with the clinical assessment (prompted by the 18-point check list). The discriminative performance of these two methods, in terms of area under the ROC curve (AUC), is presented in Table 5. Prediction was compared over 30, 60, 90, and 180 days. The clinician prediction had relatively low predictive ability with AUCs of 0.55 to 0.59 over the four time points when predicting high-risk events. AUCs for the EMR model were consistently better, ranging from 0.73 to 0.79. Similar differentials were also observed when predicting either moderate or risk events, where the AUCs were in the range 0.52 - 0.54 for clinicians and 0.71 - 0.79 for EMR models.

**Table 5:** Area under the ROC curve (AUC, 95% CIs) of clinicians versus EMR-based model on the validation cohort

		**Clinician**	**EMR model**
***High-risk versus the rest***			
	*30 days*	0.55 (0.44, 0.67)	0.73 (0.62, 0.84)
	*60 days*	0.59 (0.50, 0.69)	0.79 (0.70, 0.85)
	*90 days*	0.58 (0.50, 0.66)	0.79 (0.72, 0.84)
	*180 days*	0.57 (0.49, 0.63)	0.75 (0.69, 0.80)
***Moderate/high-risk versus low-risk***			
	*30 days*	0.52 (0.46, 0.67)	0.71 (0.64, 0.77)
	*60 days*	0.54 (0.49, 0.59)	0.78 (0.73, 0.82)
	*90 days*	0.53 (0.49, 0.58)	0.79 (0.75, 0.83)
	*180 days*	0.53 (0.49, 0.57)	0.76 (0.73, 0.80)

In identifying high risks within 90 days, clinicians achieved sensitivity of 0.08 (95% CIs: 0.02-0.2) at specificity of 0.97 (95% CIs: 0.96-0.98). The EMR model, on the other hand, reached sensitivity of 0.28 (95% CIs: 0.16-0.41) at the same specificity. Note that this low sensitivity was due to unusually high specificity of 0.97. At specificity of 0.72 (95% CIs: 0.70-0.73) the EMR model had sensitivity of 0.70 (95% CIs: 0.56-0.83). For either moderate or high-risks, clinicians scored 0.27 (95% CIs: 0.19-0.35) sensitivity and 0.82 (95% CIs: 0.81-0.84) specificity. The scores for the EMR model were higher at specificity of 0.58 (95% CIs: 0.50-0.66) at the same specificity.

Of two patients in the validation cohort who completed suicide during 30 days of follow-up, neither was identified as high-risk by clinicians while one was categorised as high-risk by the EMR model. Three patients completed suicide within 90 days. None were identified as high-risk by clinicians while the EMR model correctly classified two of the three patients.

### Risk factors

Table 6 shows the relative importance of the top factors that are predictive of high-risk events in 90 days following suicide assessment. Prior high-risk events, injuries and poisoning were indicative of subsequent high-risk events. Mental health male patients who moved homes (as approximated by postcode change) were also at risk. These risk factors were related but different from those associated with subsequent moderate-risk events (Table 7). For example, prior emergency visits, addictive drugs treatment and moderate-risk events were predictive of the future moderate-risk but not the high-risk.

**Table 6:** High-risk predictive factors in the EMR model for 3-month prediction

**Risk factor**	**Relative importance**	**Wald statistic**	**Selection probability**	**Coefficient: 95% CIs**
High-risk events past 3 months	52.3	1.4	0.92	(0.00, 0.99)
Injuries from other activities past 3–6 months	48.9	1.4	0.89	(0.00, 0.90)
Male & postcode changes past 3–6 months	37.5	0.9	0.72	(0.00, 0.96)
Poisoning by psychotropic drugs past 3 months	36.6	1.3	0.9	(0.00, 1.02)
Injuries from other activities past 3–6 months	25.9	0.8	0.65	(0.00, 0.65)
High-risk events past 24–48 months	25.4	0.8	0.65	(0.00, 0.78)
High-risk events past 3–6 months	21.1	0.9	0.74	(0.00, 0.76)
Near-misses past 3 months	17.6	0.8	0.71	(0.00, 0.64)

MHDG: Mental Health Diagnosis Group.

**Table 7:** Moderate-risk predictive factors in the EMR model for 3-month prediction

**Risk factor**	**Relative importance**	**Wald statistic**	**Selection probability**	**Coefficient: 95% CIs**
Emergency visits past 3 months	100.0	3.9	1.00	(0.60, 1.83)
Number of mental health diagnosis groups	52.7	2.2	0.99	(0.08, 1.35)
Problems related to lifestyle past 12–24 months	29.7	1.5	0.91	(0.00, 1.20)
Personal history of risk-factors past 6–12 months	23.4	1.8	0.98	(0.00, 1.36)
Male & postcode changes past 3 months	21.9	1.1	0.82	(0.00, 1.04)
Moderate-risk events past 6–12 months	20.4	1.3	0.93	(0.01, 0.99)
Problems related to lifestyle past 6–12 months	17.3	1.1	0.83	(0.00, 0.99)
Moderate-risk events past 3 months	17.0	1.1	0.92	(0.01, 0.88)
Poisoning by unspecified drugs, medicaments and biological substances past 3 months	16.7	1.1	0.84	(0.00, 1.15)
Symptoms and signs involving emotional state past 3 months	15.5	1.2	0.86	(0.00, 0.86)
Mental health diagnoses: depressive episodes; bipolar disorders past 3 months	15.3	1.0	0.76	(0.00, 1.05)
Drugs used to treat addictive disorders past 3 months	15.1	1.2	0.86	(0.00, 1.73)

MHDG: Mental Health Diagnosis Group.

## Discussion

We have developed a robust predictive model that takes information rich administrative EMR data and stratifies mental health a patient into three ordinal categories: low, moderate and high-risks of suicidal behaviours in 30, 60, 90 and 180 days following a risk assessment. For comparison against the common practice of assessment by clinicians, we validated an 18-point checklist developed and used at Barwon Health. The EMR model showed much greater predictive ability than clinicians using the checklist in identifying high-risk patients.

We followed 7,399 consecutive patients judged to be at risk of suicide for 180 days following a clinical assessment. Although this time frame is considerably short for rare events such as suicides, this was chosen for practical purposes in treatment and resources planning. Our first aim was to document risk factors associated with high and moderate lethality behaviours. We have confirmed that high lethality suicidal behaviour is associated with social and demographic factors and prior high-risk events of injuries and poisoning. On the other hand, moderate lethality behaviour is related to prior interactions with health services, history of moderate-risk events, mental disorders and lifestyle problems. These results are in line with previous studies, in that the number and potential lethality of previous suicide attempts predict lethality of future attempts [9,14]. Also, a range of mental health diagnoses were, as previously shown, predictive of future attempts during follow-up [10-12]. Social factors such as postcode change are strongly associated with suicide risk, concordant with recent findings [13,33]. We document that 66.8% of patients undergoing suicide assessment attended ED and that 41.8% of patients had been admitted to hospital in the year before their assessment. The majority of these encounters were not for episodes of self-harm or even for mental health problems. This information added stimulus to exploring use of administrative EMR data in risk stratification.

Our second aim was to examine the contribution of mental and physical health diagnoses to suicide risk. It appears that mental health diagnoses are powerful predictors of moderate-risk behaviour. However, medical conditions also contribute to risk. Previous studies have confirmed that hospital admission for non-psychiatric conditions are associated with increased suicide risk [23,24].

The next aim was to validate the 18-point risk checklist. Most of the factors in the checklist were more prevalent in patients with higher risk. To verify that if the effectiveness of checklist could be improved further, we tested same statistical framework in this paper on predictors derived from the 18 aspects and demographic data. The results were very encouraging – the algorithm outperformed the clinician’s overall risk assignment, and the difference was statistically significant. We conclude, therefore, that the risk checklist is a valid decision support tool. Although there has been some pessimism about the ability to predict suicide risk in the clinical setting, [18,19] recent reports of predictive models suggest that clinically useful risk stratification is possible using recognised risk factors [15-17].

Our fourth aim was to document the predictive performance of clinicians prompted by the 18-point risk checklist. This was relatively poor, underlining the potential utility of a decision support tool. A recent study documented the potential for clinical decision rules to augment clinical performance in risk stratification of patients who might be at risk of suicide [34]. The models studied had high sensitivity but low specificity, unlike those reported in our study which had lower sensitivity but high specificity. Recent systemic studies of the factors associated with high risk in suicide-prone patients have added to knowledge on the potential contribution of known risk factors to prognostic stratification, and may help in the design of effective decision support algorithms [35,36].

Our final aim was to use predictive models applied to data from the EMR to derive predictive models for high-risk suicide behaviour. Models for prediction of high-risk behaviour used administrative, social and demographic, and clinical data. Risk stratification using these models was more accurate than clinical stratification. The potential of EMR data to identify patients at high-risk of suicide from overdose has been documented in a recent study of paediatric patients [37].

The strengths of this study include the large size of the dataset, the fact that patients were consecutively recruited from a defined geographical region, and the comprehensive follow-up. Suicide assessments in both hospital and community settings were included. The models based on EMR could be updated in real-time, and make use of data that are routinely collected. The predictors derived from the EMR data were standardised, and thus the tools can be generalisable to sites with similar EMR systems. Limitations are that it is a retrospective study confined to a single location. The predictive models were validated in the setting in which they were derived but their performance may differ in other settings. The problem studied is inherently difficult to predict and there may be limits to the performance of predictive models due to the fact that individual factors are only weakly predictive.

## Conclusions

In conclusion, demographic, social, mental and physical health, and administrative data related to health service encounters can all be used to identify patients at risk of self-harm behaviour. A history of high risk events are indicative of short-term future high-lethality suicide attempts. Clinicians’ assessment could be enhanced by using data from a simple checklist of known risk factors. Modern statistical techniques applied to complex data from a comprehensive EMR has considerable potential to improve risk stratification of patients that may engage in repeated self-harm attempts.

## Abbreviations

AUC: Area under the ROC Curve; CI: Confidence interval; ED: Emergency department; EMR: Electronic medical record; ICD-10: International statistical classification of diseases and related health problems 10th revision; ROC: Receiver operating characteristic.

## Competing interests

MB has received Grant/Research Support from the NIH, Cooperative Research Centre, Simons Autism Foundation, Cancer Council of Victoria, Stanley Medical Research Foundation, MBF, NHMRC, Beyond Blue, Rotary Health, Geelong Medical Research Foundation, Bristol Myers Squibb, Eli Lilly, Glaxo SmithKline, Meat and Livestock Board, Organon, Novartis, Mayne Pharma, Servier and Woolworths, has been a speaker for Astra Zeneca, Bristol Myers Squibb, Eli Lilly, Glaxo SmithKline, Janssen Cilag, Lundbeck, Merck, Pfizer, Sanofi Synthelabo, Servier, Solvay and Wyeth, and served as a consultant to Astra Zeneca, Bristol Myers Squibb, Eli Lilly, Glaxo SmithKline, Janssen Cilag, Lundbeck Merck and Servier.

## Authors’ contributions

TT implemented the prediction model. RH and SV conceived of the study. WL performed the data extraction. TT, WL, DP, and SV carried out the statistical analysis. RH, MB, RK and SV participated in coordination. All authors participated in the design of the study, helped to draft the manuscript. All authors read and approved the final manuscript.

## Pre-publication history

The pre-publication history for this paper can be accessed here:

http://www.biomedcentral.com/1471-244X/14/76/prepub
